# Exploring Early Complications in Paraumbilical Hernia Mesh Repair: A Rigorous Six-Month Prospective Study and In-Depth Analysis

**DOI:** 10.7759/cureus.73348

**Published:** 2024-11-09

**Authors:** Niamat Ullah, Gul Sharif, Muhammad Salman, Sajjad Muhammad Khan Shinwari, Qazi Kamran Amin

**Affiliations:** 1 General Surgery, Medical Teaching Institute, Lady Reading Hospital, Peshawar, PAK; 2 General Surgery, Lady Reading Hospital, Peshawar, PAK; 3 General Surgery, Jinnah Medical and Dental College, Peshawar, PAK; 4 General Surgery, Institute of Kidney Disease, Peshawar, PAK; 5 General Medicine, Rehman Medical Institute, Peshawar, PAK

**Keywords:** hematoma, mesh hernioplasty complications, mesh repair, post-operative seroma, surgical wound infection

## Abstract

Introduction

When an organ, such as the colon, pushes through the wall of the abdominal cavity, a hernia results. After femoral and inguinal hernias, umbilical hernias account for the third most common kind of abdominal hernia in adults precipitated by conditions such as obesity, ascites, and repeated pregnancies. A subtype of umbilical hernias called paraumbilical hernias is more likely to cause problems such as rupture, skin ulceration, and obstruction. Seroma, hematoma, and infection are the reported post-repair consequences but data regarding early complications is limited. High-quality data assessing early complications is necessary to improve mesh repair outcomes.

Materials and methods

This cross-sectional study was carried out in the Department of General Surgery at Medical Teaching Institute (MTI) Lady Reading Hospital, Peshawar over one year, from January to December 2022. A total of 167 patients were selected using simple random sequential selection. Patients aged 20-60 years of both genders who were diagnosed with paraumbilical hernia in the emergency department were included. To prevent bias, those with uncontrolled diabetes or existing complications from hernia were excluded. Following informed consent, data were gathered using pre-designed proformas. Patients underwent open mesh repair during each surgery, and they were monitored at one and three months following the procedure. Complications such as seroma, hematoma, and wound infection were documented. Data were analyzed using SPSS version 20 (IBM Corp., Armonk, NY) with chi-square tests for categorical variables and a significance level of p < 0.05.

Results

The study included a total of 167 patients, with a mean age of 42 years (SD ±8.77). The majority of patients (40%) ranged in age from 41 to 50 years old, with 33% aged 31 to 40. Gender distribution revealed that 63 (38%) of the patients were male and 104 (62%) were female. Early complications included 25 (15%) wound infections, 32 (19%) seromas, and 63 (38%) hematomas. The occurrence of wound infections, seromas, and hematomas did not differ significantly by age or gender (p > 0.05).

Conclusion

Early complications from paraumbilical hernia mesh repair include wound infections (15%), seromas (19%), and hematomas (38%). Postoperative monitoring is critical to reducing these complications.

## Introduction

Hernias, characterized by the protrusion of organs through weakened abdominal walls, present a prevalent surgical challenge. Paraumbilical hernias occur due to a weakness in the abdominal wall around the umbilicus or navel where the umbilical cord would pass [1]. After inguinal and femoral hernias, the umbilical type of abdominal hernias is the third most common in adults, accounting for 3% to 8.5% of cases [2]. Large abdominal tumors, obesity, ascites, repeated pregnancies, and other diseases that weaken the fascia covering the umbilicus and increase intra-abdominal pressure are frequently linked to the formation of umbilical hernias. The European and American Hernia Societies categorize adult umbilical hernias based on size. A small hernia is defined as less than 1 cm in diameter, a medium hernia ranges from 1 to 4 cm, and a large hernia exceeds 4 cm [3,4].

The detection of hernias may occur during routine physical examinations, with patients either presenting asymptomatically or due to complications associated with the hernia [5]. Asymptomatic hernias usually appear as congestion or edema at the site of hernia, sometimes with radiating pain into the surrounding tissue. These hernias, in contrast to symptomatic hernias, are defined by the lack of actual discomfort or soreness on inspection and grow with standing or increased intra-abdominal pressure [6].

Umbilical hernia repair is a prevalent surgical intervention; nonetheless, it is not without complications. Seroma, hematoma, wound infection, intestinal injury, paralytic ileus, and hernia recurrence are among the well-documented early postoperative complications [7]. Of these, wound infection is especially dangerous since it can have a major effect on the course of treatment for the patient, resulting in a longer recovery period, recurrence, and unwanted scarring [8]. In order to improve patient outcomes and guarantee the success of hernia repair, this issue must be addressed.

The risk of wound infection is increased with mesh repair. Though mesh is widely used to reinforce the abdominal wall, it adds another level of complexity to infection control [9]. Understanding the causes, frequency, and treatment of mesh-related infections is critical for improving surgical outcomes. To develop effective techniques for reducing mesh infections and improving long-term outcomes in ventral incisional hernia (VIH) repairs, high-quality reporting is required, supported by observational studies and prospective trials [10]. A systematic focus on early complications, particularly in paraumbilical hernia repair, has the potential to result in timely detection and intervention [11]. By addressing these concerns early, healthcare providers can not only prevent complications from progressing but also lower the total burden on patients and the healthcare system [12,13].

This study aims to evaluate the incidence of early complications following mesh repair of paraumbilical hernias, with a focus on wound infections. The findings will contribute to the development of evidence-based guidelines for managing and preventing these complications, thereby improving the overall success of hernia repair surgeries.

## Materials and methods

This study was carried out from January to December 20, 2022 in the Department of General Surgery at the Medical Teaching Institute (MTI) Lady Reading Hospital, Peshawar. Based on a 19% infection rate in post-mesh repair paraumbilical hernia, a sample size of 167 patients was determined using openepi.com with a 90% power and 5% significance level. The participants were chosen using simple random consecutive sampling. Patients of both sexes aged 20 to 60 years or older who were diagnosed with paraumbilical hernia and admitted through the outpatient department (OPD) met the inclusion criteria. In order to prevent confounders and bias in the study outcomes, we excluded the following patients: those who were hospitalized after visiting the emergency room; individuals with complex paraumbilical hernias such as gangrenous, obstructed, and strangulated hernias; patients with recurrent hernias; and those with comorbidities such as uncontrolled diabetes.

Data collection procedures

The IRB of Lady Reading Hospital approved and issued the IRB approval number IRB/LRH/MTI/2022/388 for the study. After obtaining informed written consent, patients visiting the OPD were recruited for the study. Personal information was collected using a pre-designed proforma. Any indications of infection or inflammation in the hernia were assessed. After being admitted, patients underwent testing for standard investigations. Surgeons and staff who specialized in open mesh treatment of paraumbilical hernias performed all of the surgeries. All surgeons carrying out the surgeries adhered to a predetermined protocol in order to minimize variation in results and standardize the surgical approach among various practitioners. This covered standardized procedures for postoperative care, suture methods, and mesh implantation. To guarantee consistency between cases, the surgical team was also briefed on these methods before the research period began.

The hernial sac was excised during this treatment. Underneath, where the hernia was located, the mesh was placed. Sutures were placed into the stronger tissue surrounding the hernia and were used to connect the polypropylene mesh. The mesh was stretched 3 to 4 centimeters past the hernia's margins. The muscle was secured to the umbilicus once more. Following their recuperation, all patients were advised to follow up at one- and three-month marks following surgery.

According to operational criteria, patients were screened for three primary complications: surgical wound infection, seroma, and hematoma. Specific criteria and definitions were used to guarantee uniformity in the evaluation of postoperative complications. The CDC's criteria for surgical site infections, which include symptoms such as pain, purulent discharge, and erythema, were used to assess wound infections. The presence of a fluid collection at the surgical site identified either clinically or by ultrasonography in the early postoperative phase was referred to as seroma formation. Clinical examination criteria, such as localized swelling, ecchymosis, and discomfort at the surgical site, were used to identify hematomas. Accurate reporting of complications across cases and consistent follow-up evaluations were made possible by these defined standards [14].

Data analysis

Statistical analysis of data was performed using SPSS software version 20 (IBM Corp., Armonk, NY). Descriptive analysis was performed using mean and standard deviation for continuous variables such as age; frequencies and percentages were used for categorical variables like age groups, gender, and early complications including wound infection, seroma, and hematoma. These variables were stratified by age and gender. The chi-square test was applied for categorical variables. P-value ≤ 0.05 was considered significant.

## Results

**Figure 1:**
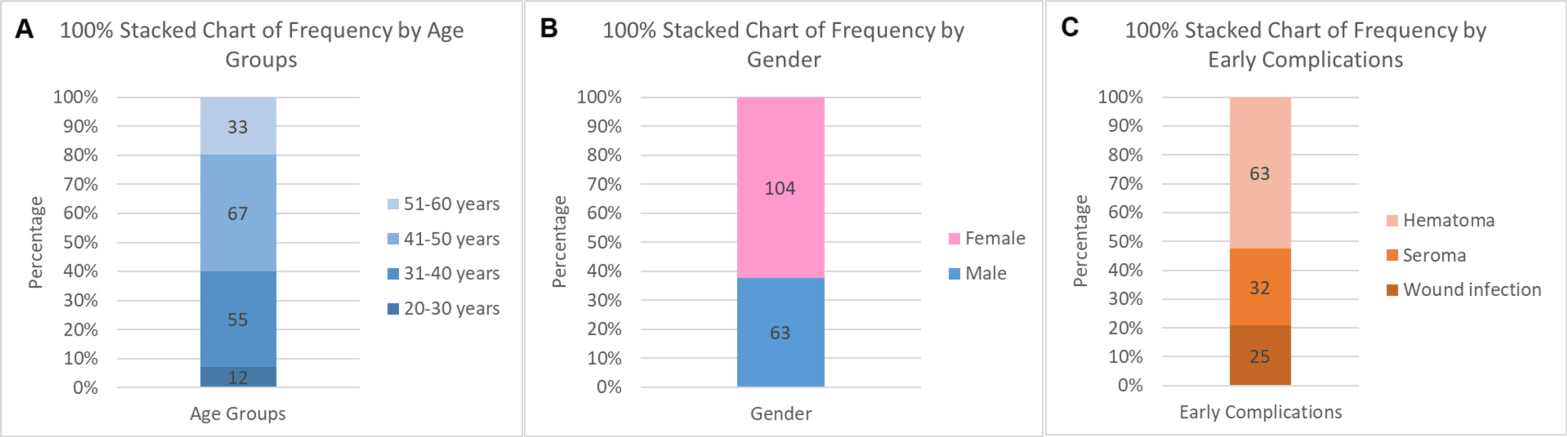
Graphical representation of the distribution of the categorical variables (N=167), A: Frequency Distribution of Age Groups, B: Frequency Distribution of Gender, C: Frequency Distribution of Early Complications Mean age was 42 years with SD ±8.77

This study aimed to measure the frequency of early problems in 167 individuals receiving mesh treatment for paraumbilical hernia. The cohort's age distribution revealed that, with a mean age of 42 years (SD ±8.77), 12 patients (7%) were between the ages of 20 and 30; 55 (33%) were in the 31-40 range; 67 (40%) were in the 41-50 range; and 33 (20%) were between the ages of 51 and 60. The patients' gender distribution showed that 104 (62%) were female and 63 (38%) were male. According to the analysis of early complications, there were 63 patients (38%), 32 patients (19%), and 25 patients (15%) who experienced a hematoma, seroma, or wound infection (Figure 1).

Stratification of complications by age (Table 2) indicated no statistically significant differences in the occurrence of wound infection (p = 0.7815), seroma (p = 0.9922), or hematoma (p = 0.9856) among age groups.

**Table 1:** Stratification of early complications with age P-Value less than 0.05 was considered significant using Chi-square test.

Early Complications		21-30 years	31-40 years	41-50 years	51-60 years	Total	Chi-Square Value	P Value (Chi-Sq)
Wound infection	Yes	3	8	9	5	25	1.08	0.7815
	No	9	47	58	28	142		
Seroma	Yes	2	11	13	6	32	0.970	0.9922
	No	10	44	54	27	135		
Hematoma	Yes	4	21	25	13	63	0.147	0.9856
	No	8	34	42	20	104		

Similarly, the stratification of early complications by gender (Table 3) did not indicate any significant differences for wound infection (p = 0.7990), seroma (p = 0.9767), or hematoma (p = 0.9386) between male and female patients. 

**Table 2:** Stratification of early complications with gender P-value less than 0.05 was considered significant using chi-square test.

Early complications		Male	Female	Total	Chi-square value	P-value (chi-square)
Wound infection	Yes	10	15	25	0.065	0.7990
	No	53	89	142		
Seroma	Yes	12	20	32	0.001	0.9767
	No	51	84	135		
Hematoma	Yes	24	39	63	0.006	0.9386
	No	39	65	104		

## Discussion

Our investigation revealed a diverse age distribution within the studied 167 patients: 7% fell within the 20-30 age bracket, 33% within 31-40 ages, 40% within 41-50 ages, and 20% within 51-60 ages. The mean age stood at 42 years, showcasing a standard deviation of ±8.77. Gender distribution reflected 38% male and 62% female representation. Early complications after mesh repair of paraumbilical hernias are common, including hematoma (38%), seroma (19%), and wound infection (15%). To determine demographic implications on paraumbilical hernia repair complications, age, and gender correlations were examined. Understanding these links is important since significant correlations may need patient group-specific preventive interventions. Our findings were not statistically significant, suggesting that clinical factors rather than age or gender influence early complications like wound infections, seromas, and hematomas. Clinical practice benefits from this discovery because it supports standardized postoperative treatment across varied demographic groups without age or gender-specific changes.

These findings highlight the necessity of standardizing preventive measures for all patient demographics and of conducting close postoperative monitoring in order to reduce the likelihood of early problems following paraumbilical hernia repair, thus reducing the possibility of readmissions and disease burden on the hospital. A study by Bittner involving 3,431 operations on 3,383 patients found that 5.3% of patients required readmissions at 30 days, predominantly due to wound-related issues, seromas, and pain. The study reported a median hospital stay of 0-1 day, with complication and mortality rates of 4.1% and 0.1%, respectively [15].

Concurring results emerged from Malik et al.'s study, where wound infection dominated, posing challenges due to its potential impact on mesh integrity; seromas were minimal at 1%, while hematomas were recorded at 2% [16]. Daudpoto et al. identified varying postoperative complications, reporting wound infections in 15% of patients in group A and 10% in group B, which were treated conservatively [17]. Girometti et al. found comparable wound infection rates of 10%, challenging the notion that mesh utilization independently heightens infection risks [18]. Ponten et al.'s studies reported higher wound infection rates when mesh was employed, contrary to observations in our study. However, their findings aligned concerning hematoma and seroma occurrences, prevalent in patients not drained and subjected to classical Mayo's repair [19].

Musilova et al.'s 2016, study further illuminated the advantages of laparoscopic techniques, with significantly lower wound infection rates (5.64%) compared to open surgical approaches (18.91%). This observation corroborated with other studies, collectively advocating for a substantial reduction in wound infections through laparoscopic ventral hernia repair. The implications of these findings underscore the intricate landscape of complications in hernia repairs, necessitating a nuanced approach to optimize patient outcomes [20].

Wound infections, seromas, and hematomas can be reduced using new postoperative care methods. Best practices emphasize perioperative antibiotic prophylaxis, particularly with broad-spectrum antibiotics, to limit infection risk in mesh repair procedures. This method has been found to minimize surgical site infections by roughly 30% in abdominal hernia repairs [21]. Additionally, rigorous intraoperative handling of tissues and strict adherence to aseptic methods are critical to preventing infections and seroma development [22]. Negative pressure wound therapy (NPWT) has also shown success in increasing wound healing and minimizing hematomas and seromas, notably in abdominal and hernia procedures, by maintaining a controlled wound environment [23]. These approaches, when paired with early postoperative monitoring and mobilization, contribute significantly to minimizing complications and improving patient outcomes [24].

The present study has a number of limitations. The 167-patient sample size and single-center methodology limit how far the findings can be generalized. Understanding long-term consequences like hernia recurrence may also be hampered by factors such as the exclusion of patients with comorbidities like diabetes and the short follow-up time. Further factors that might have affected complication rates were differences in surgical methods and postoperative care, which were not fully taken into consideration. These results need to be confirmed by larger multicenter cohort studies with longer follow-up periods.

## Conclusions

This study emphasizes how common early problems including seromas, hematomas, and wound infections can be when mesh treatment is used for paraumbilical hernias. Our findings highlight the significance of careful postoperative surveillance and early management in reducing complications by identifying these risks. This study aims to further the development of recommendations meant to enhance surgical safety and patient outcomes. To confirm these findings and investigate long-term issues such as hernia recurrence, multicenter studies with larger sample sizes and longer follow-up times are advised.
